# Granulomatous interstitial nephritis in a patient with SARS-CoV-2 infection

**DOI:** 10.1186/s12882-020-02213-w

**Published:** 2021-01-08

**Authors:** Katarzyna Szajek, Marie-Elisabeth Kajdi, Valerie A. Luyckx, Thomas Hans Fehr, Ariana Gaspert, Alexia Cusini, Karin Hohloch, Philipp Grosse

**Affiliations:** 1 Department of Critical Care, Cantonal Hospital Graubuenden, Chur, Switzerland; 2 Department of Internal Medicine, Division of Nephrology, Cantonal Hospital Graubuenden, Chur, Switzerland; 3 Department of Pathology and Molecular Pathology, University Hospital Zurich, Zurich, Switzerland; 4 Department of Internal Medicine, Division of Infectious Diseases, Cantonal Hospital Graubuenden, Chur, Switzerland; 5 Department of Internal Medicine, Division of Oncology/Hematology, Cantonal Hospital Graubuenden, Chur, Switzerland; 6 Department of Hematology and Oncology, Georg August University, UMG, Goettingen, Germany

**Keywords:** COVID-19, Acute kidney injury, Hemolysis, Granulomatous interstitial nephritis, Corticosteroids, Case report

## Abstract

**Background:**

Acute kidney injury (AKI) associated with severe coronavirus disease 19 (COVID-19) is common and is a significant predictor of morbidity and mortality, especially when dialysis is required. Case reports and autopsy series have revealed that most patients with COVID-19 – associated acute kidney injury have evidence of acute tubular injury and necrosis - not unexpected in critically ill patients. Others have been found to have collapsing glomerulopathy, thrombotic microangiopathy and diverse underlying kidney diseases. A primary kidney pathology related to COVID-19 has not yet emerged. Thus far direct infection of the kidney, or its impact on clinical disease remains controversial. The management of AKI is currently supportive.

**Case Presentation:**

The patient presented here was positive for SARS-CoV-2, had severe acute respiratory distress syndrome and multi-organ failure. Within days of admission to the intensive care unit he developed oliguric acute kidney failure requiring dialysis. Acute kidney injury developed in the setting of hemodynamic instability, sepsis and a maculopapular rash. Over the ensuing days the patient also developed transfusion-requiring severe hemolysis which was Coombs negative. Schistocytes were present on the peripheral smear. Given the broad differential diagnoses for acute kidney injury, a kidney biopsy was performed and revealed granulomatous tubulo-interstitial nephritis with some acute tubular injury. Based on the biopsy findings, a decision was taken to adjust medications and initiate corticosteroids for presumed medication-induced interstitial nephritis, hemolysis and maculo-papular rash. The kidney function and hemolysis improved over the subsequent days and the patient was discharged to a rehabilitation facility, no-longer required dialysis.

**Conclusions:**

Acute kidney injury in patients with severe COVID-19 may have multiple causes. We present the first case of granulomatous interstitial nephritis in a patient with COVID-19. Drug-reactions may be more frequent than currently recognized in COVID-19 and are potentially reversible. The kidney biopsy findings in this case led to a change in therapy, which was associated with subsequent patient improvement. Kidney biopsy may therefore have significant value in pulling together a clinical diagnosis, and may impact outcome if a treatable cause is identified.

## Background

Acute kidney injury (AKI) associated with severe coronavirus disease 19 (COVID-19) is common and is a significant predictor of morbidity and mortality, especially when dialysis is required [1–6]. Case reports and autopsy series have revealed that most patients with COVID-19-associated AKI have evidence of acute tubular injury (ATI) and/or acute tubular necrosis (ATN) - not unexpected in critically ill patients [7–9]. A mild associated interstitial infiltrate may be present [10]. Other biopsy findings have included collapsing glomerulopathy (associated with African ancestry and a high-risk APOL1 genotype [11, 12], thrombotic microangiopathy, and diverse underlying kidney diseases [8, 13]. Kidney infarction has also been reported [14]. A primary kidney pathology related to COVID-19 has not yet emerged. Thus far direct infection of the kidney remains controversial [8, 10, 13]. Recent description of viral particles in the tubular epithelium may support this possibility, although the clinical significance of this remains unknown [15].

At present, the management of AKI is supportive. During the first wave of SARS Cov2, around 1 in 4 patients with severe COVID and intubated the intensive care unit (ICU) require dialysis [6, 16]. Mortality rates are higher in patients with hospital-acquired AKI compared with community-acquired AKI associated with COVID-19 [4]. Ongoing vigilance is therefore required throughout the hospital course. Many patients, given the severity of illness, receive multiple medications including a variety of antibiotics, and increasingly potential therapies are being tested with encouraging results. Patients may therefore be expected to be at risk of drug-associated hypersensitivity [17, 18]. Initially the use of corticosteroids was not routinely advocated, however recent data showed a reduction in 28-day mortality when used in severe COVID-19 [19]. How these therapies may impact AKI and renal recovery in patients with COVID-19 remains unknown. Here we report a patient with severe COVID-19 who had developed AKI in the setting of multiorgan dysfunction, a skin rash and hemolysis. After nephrology consultation, a kidney biopsy was performed, which led to a change in management and patient improvement.

## Case presentation

A 62-year-old Caucasian man presented with symptoms of cough, fever, myalgia and chills. Symptoms had begun 6 days prior to admission. He had tested positive for SARS-CoV-2 by Xpert Xpress SaRS-CoV-2 (Cepheid, Dx System Version 4.8) three days after symptom onset. His past medical history was unremarkable except for hyperlipidemia treated with atorvastatin 40 mg daily. No allergies were reported, the patient did not smoke, drink alcohol or use illicit substances. Kidney function was normal on admission.

Computed tomography (CT) scan of the chest, abdomen and pelvis excluded pulmonary emboli and showed diffuse bilateral ground-glass infiltrates of the lungs with associated lymphadenopathy, moderate pleural effusions, normal-sized and -shaped kidneys with adequate perfusion and without cortical defects.

Two days after admission the patient required intubation due to acute respiratory distress syndrome (ARDS). He was managed with prone positioning and was initiated on hydroxychloroquine after exclusion of glucose-6-phosphate dehydrogenase (G6PD) deficiency. Antibiotic therapy with amoxicillin-clavulanate was given empirically assuming bacterial superinfection of viral pneumonia. His clinical condition worsened with the development of atrial fibrillation, AKI, paralytic ileus, hemolytic anemia and a maculopapular rash on the trunk and lower extremities.

**Fig. 1:**
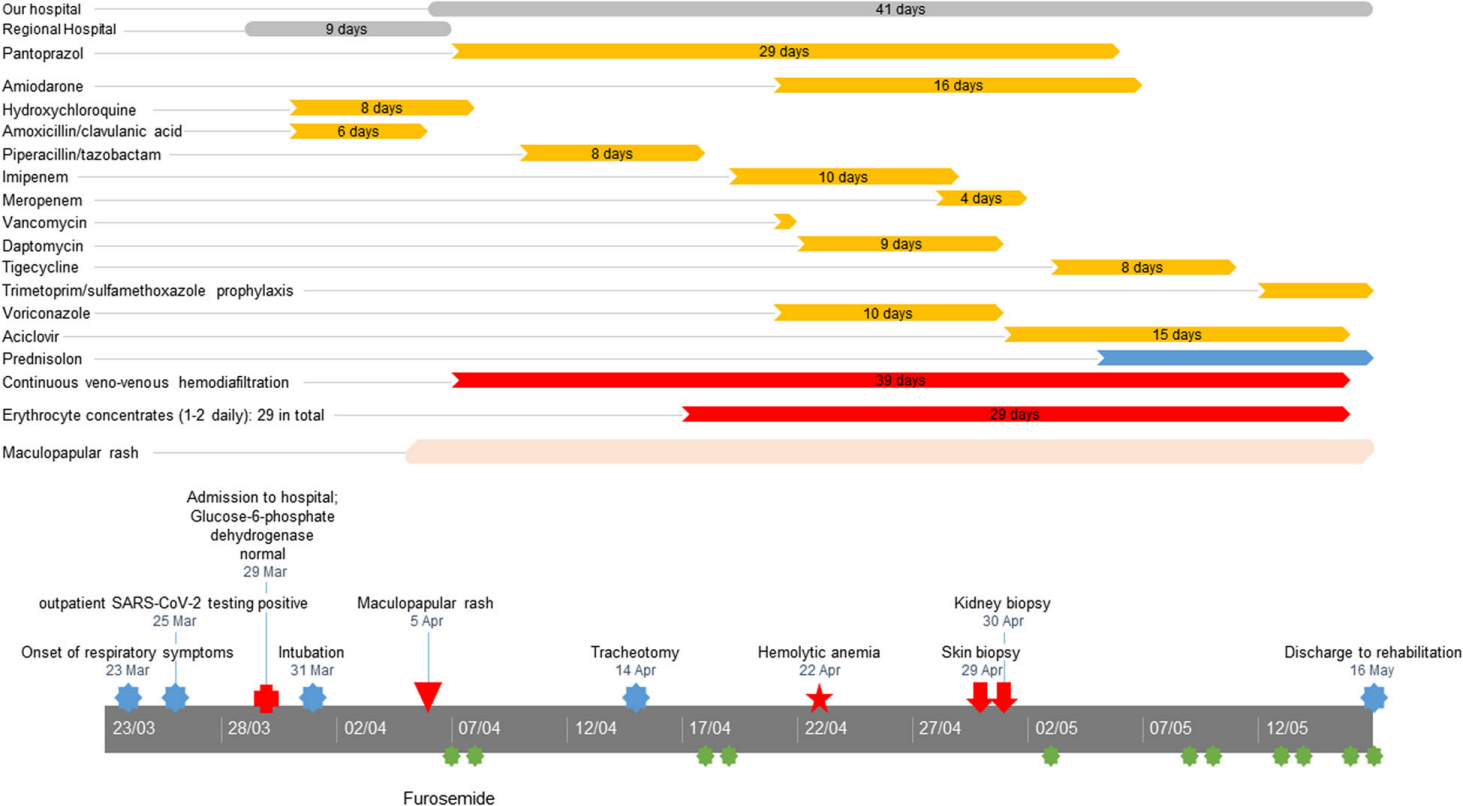
Timeline

The chronologic sequence of medications and clinical events are highlighted in Fig. 1. Laboratory results are shown in Table 1. Details of affected organ systems, diagnostics and therapies are listed in Table 2.

**Table 1:** Laboratory results

Laboratory Test	Reference range	Admission to hospital (Day 0)^a^	Day of transfer to tertiary center (Day 8) ^a^	Hemolytic anemia (Day 24)^a^	Renal consult (Day 26)^a^	Day of Biopsy (Day 32)^a^	Day of transfer (Day 48)^a^
**BLOOD ANALYSIS**							
Urea, mmol/l	2.76–8.07	-	37.4	CVVHD	CVVHD	CVVHD	29.8
Creatinine, umol/l	59–104	87	498	CVVHD	CVVHD	CVVHD	130
Albumin, g/l	39.7–49.5	-	18.5	19.1	25.8	19.8	22.7
CRP, mg/l	< 5	223	310	339	150	201	27.1
PCT, ng/ml	< 0.5	0.29	2.42	9.13	3.71	5.05	2.56
Ferritin, ug/l	30–400	-	> 11,063	-	2505	4646	3480
D-dimer, mg/l	< 0.5	1.38	3.98	2.55	2.77	2.63	-
IL-6, pg/ml	< 7	-	-	-	-	56.2	-
AST, U/l	< 40	60	252	49	68	73	38
ALT, U/l	< 50	78	146	43	35	47	78
Bilirubin totally, umol/l	3.4–17	-	15.1	29.4	17.3	24	-
Bilirubin indirect, umol/l	< 12.8	-	0.9	3.9	-	-	-
Hemoglobin, g/l	140–180	138	91	69	72	78	71
Schistocytes	-			+		+	
Platelet count	139–335 10E3/ul	241	612	598	501	298	513
Haptoglobin, g/l	0.3-2.0	-	-	< 0.1	< 0.1	< 0.1	0.91
LDH, U/l	240–480	770	999	1173	1125	1109	636
Coombs test	Positive/negative	-	-	-	negative	-	-
WBC count	3.5–10 10E3/ml	10	8.6	36.5	25.8	23.8	12.9
Eosinophils	0.08–0.36 10E3/ul	0.01	0.11	0.51	0.12	1.10	0.68
**URINE ANALYSIS**							
Fractional excretion of Urea (%)		-	46.8	-	-	-	-
Urine PCR, mg/mmol	< 20	-	72.6	-	-	-	-
Urine ACR, mg/mmol	< 3	-	5.3	-	-	-	-
Urine, red blood cells, /ul	< 23	10	388	-	13.6	829	3 (03.05.)
Urine, leucocytes, /ul	< 25	15	8	-	5.1	29.1	1 (03.05)

Abbreviations: *CRP* C-reactive proteine, *PCT* procalcitonin, *IL-6* interleukin 6, *AST* aspartate amino transferase, *ALT* alanine aminotransferase, *LDH* lactate dehydrogenase, *WBC* white blood cells, *PCR* protein/creatinine ratio, *ACR* albumine/creatinine ratio

^a^+/- 3 days

**Table 2:** Affected organ systems and therapeutic measures

Affected organ system / Medical problem	Diagnostics / Results	Therapy
Severe acute respiratory distress syndrome (ARDS) with PaO2/FiO2 ratio as deep as 80	CT scan thorax / bilateral ground-glass infiltrates of the lungs, pleural effusions	Prone positioning Nitric oxide therapy
Co-infections causing pneumonia and sepsis	- ventilator associated pneumonia with Proteus vulgaris and sepsis - viral pneumonia with Herpes simplex virus 1 - catheter infection with Staphylococcus epidermidis	Thoracic drain Antimicrobial therapy
Acute kidney injury (AKI)	Kidney biopsy / granulomatous tubulointerstitial nephritis	Continous veno-venous hemodiafiltration Discontinuation of beta-lactams & proton pump inhibitor Corticosteroid therapy
Encephalopathy	- CT and MRI head / multiple intracranial microhemorrhages - EEG/ no epileptic activity	Termination of unnecessary medication Temporary reduction of anticoagulation Physiotherapy
Hemodynamic instability	ECG / Intermittent atrial fibrillation Echocardiography / left ventricular function within normal limits	Vasopressors Amiodarone Electric cardioversion Therapeutic anticoagulation
Hemolytic anemia	Laboratory testing/ Coombs test negative, ADAMTS 13 normal, Blood immunophenotyping/ no evidence of paroxysmal nocturnal hemoglobinuria	Transfusion of packed red blood cells Discontinuation of imipenem and amiodarone Corticosteroid therapy
Local bleeding after tracheostomy without hemodynamic instability	Clinical examination	Transfusion of packed red blood cells Mechanical compression
Critical illness polyneuropathy	Diffuse, symmetric, flaccid paresis, muscle weakness	Physiotherapy, discharge to rehabilitation facility
Hepatopathy	Hepatitis B and C negative No cholestasis on imaging	Reduction of hepatotoxic medication
Maculopapular rash	Skin biopsy / dermoepidermal junction with focal vacuolization; lymphocytic infiltrates and rare eosinophils within the corium, discrete vasculitic changes and extravasates of erythrocytes; *consistent with drug-induced exanthema*; negative for SARS-CoV-2	Corticosteroids topically and systemically

A maculo-papular skin rash developed on day 7 after admission. Severe AKI with oliguria (AKIN 3), consecutive fluid overload and metabolic acidosis necessitated initiation of continuous veno-venous hemodiafiltration (CVVHDF) on day 9. Peak creatinine was 519 umol/L, urinalysis showed minimal proteinuria and microscopic hematuria. Proteinuria subsequently increased significantly and microscopic hematuria persisted, urine leucocytes were persistently within the normal range. **(**Table 1).

Several days after initiation of CVVHDF (on day 24) the patient developed severe microangiopathic hemolytic anemia, Coombs negative, which was transfusion dependent. Serologic screening was negative for HIV, hepatitis B and C virus infection; anti-nuclear antibodies, anti-DNA antibodies, anti-neutrophil cytoplasmic antibodies, anti-cardiolipin antibodies and complement levels were normal. Eosinophils were initially not significantly elevated. There was no evidence of urinary obstruction or rhabdomyolysis. Echocardiogram showed preserved cardiac function.

Differential diagnosis of the AKI included acute tubular injury (ATI) due to hemodynamic instability; sepsis-associated AKI; ATI with pigmented tubular casts as a consequence of hemolysis; thrombotic microangiopathy - given the ongoing severe hemolysis with schistocytes on peripheral smear (despite lack of overt thrombocytopenia); collapsing glomerulopathy - given the large rise in proteinuria,; and acute interstitial nephritis associated with antibiotics - given concurrent skin rash, although peripheral eosinophilia and leucocyturia were not marked. In the absence of improvement of kidney function a transcutaneous renal biopsy was performed while the patient was proned in ICU, 32 days after admission.

**Fig. 2:**
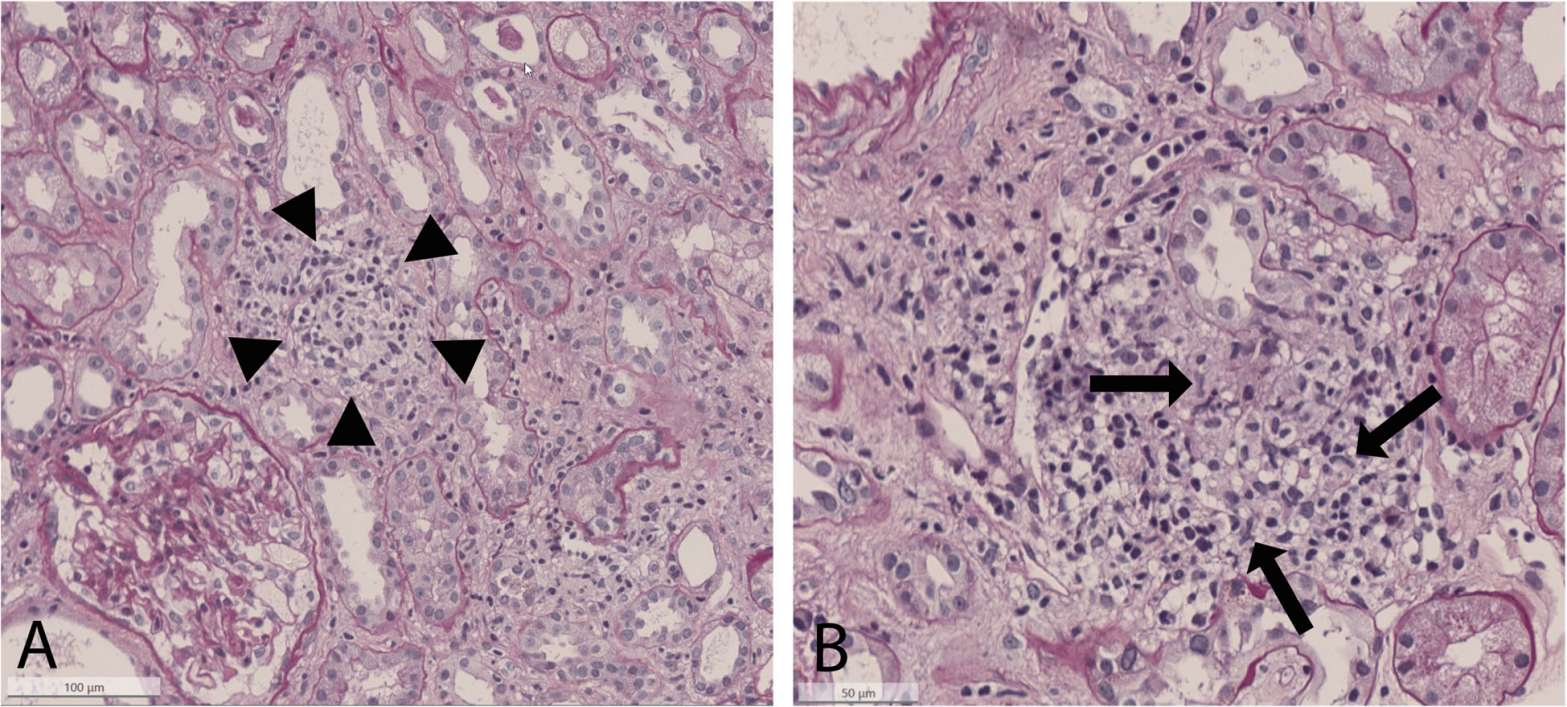
**a**: Kidney biopsy with interstitial infiltrates of mostly lymphocytes, histiocytes and plasma cells and a noncaseating granuloma (arrowheads) (PAS, Periodic acid-Schiff reaction). **b**: Detail of another peritubular granuloma with lymphocytes and epithelioid macrophages (arrows) (PAS)

Light microscopy revealed 34 mostly normal glomeruli. Few glomeruli were mildly congested, without thrombi. There was diffuse interstitial edema and focal infiltrates with lymphocytes, histiocytes, rare plasma cells, neutrophils and eosinophils. Multiple non-caseating granulomas mostly consisting of lymphocytes and epithelioid histiocytes (Fig. 2) were present. There was very mild tubulitis with rare lymphocytes in the tubular epithelium. Many tubules had a dilated lumen, flattened epithelium and loss of brush border. Some had fine, isometric vacuolization of the cytoplasm. Rare lumina contained finely granular, mostly eosinophilic and very rare brownish casts only partially positive for hemoglobin in a few tubules. Some peritubular capillaries contained mononuclear cells, but no erythrocyte aggregation. There was mild arteriolar hyalinosis and arteriosclerosis, but no thrombi or vasculitis. Immunhistochemistry showed only trace IgM, Kappa and Lambda in the mesangium. IgG, IgA, C3 and C1q were negative in the glomeruli. Electron microscopy revealed myelin figures in the cytoplasm of a few parietal epithelia. No definite viral particles were detected.

The biopsy was consistent with *granulomatous tubulointerstitial nephritis, acute tubular injury and regeneration*. There was no evidence of renal thrombotic microangiopathy, collapsing glomerulopathy or vasculitis.

Mycobacterium tuberculosis infection as excluded and confirmed by negative cultures of urine and tracheal secretions. Serology for Sjogren’s Syndrome was negative. Sarcoidosis was considered clinically unlikely, despite thoracic lymphadenopathy which was interpreted as consistent with severe SARS Cov2 pneumonia. The ionized calcium levels were normal or low during the ICU stay. Angiotensin converting enzyme and Interleukin-2 levels were however not measured. The biopsy findings could not explain the proteinuria, which was interpreted as a consequence of kidney injury and profound inflammation associated with SARS Cov2 infection.

Given that a medication reaction was a potential cause for kidney biopsy findings as well as for the rash and the hemolysis, a multidisciplinary decision was taken to stop ß-lactams, amiodarone and pantoprazole and to begin methylprednisolone 60 mg daily on day 37 (Fig. 1). 47 days after admission urine output began to improve and CVVHDF was discontinued. The hemolysis resolved, the skin rash improved.

On transfer to neurorehabilitation 48 days after admission, the patient was tetraparetic due to critical illness polyneuropathy but alert and able to follow simple commands, he had tracheostomy in place and was breathing spontaneously with little support. The course of rehabilitation showed progressive improvement of kidney function (Fig. 1). The estimated GFR two months post-discharge was 43 ml/min/1,73 m2 suggesting a likely transition to chronic kidney disease.

## Discussion and conclusions

The underlying pathophysiology of impaired kidney function in patients suffering from COVID-19 is likely complex and multifactorial and to date incompletely understood [8, 13, 20]. Virus-induced sepsis with hemodynamic instability and renal hypoperfusion may promote ATI [9, 21, 22]. Upregulation of proinflammatory cytokines and chemokines in the setting of sepsis, generally described as “cytokine storm”, may trigger multiorgan failure including ATI [20, 23, 24]; SARS-CoV-2-associated hypercoagulability may aggravate endothelial dysfunction leading to microangiopathy and collapsing glomerulopathy [25–27]. SARS-CoV-2 RNA has been isolated in urine and viral particles have been demonstrated in post-mortem kidney tissue by some authors but not others [9, 10, 15] suggesting possible renal tropism of the virus, although others have failed to find viral RNA in kidney tissue by *in-situ* hybridization or RT-PCR in kidney biopsies [28, 13]. Internalisation of coronavirus into kidney tissue may potentially be mediated through the angiotensin-converting enzyme 2 (ACE2) receptor [9, 20, 29].

We report a case of GIN in a patient with COVID-19 who required prolonged CVVHDF. Clinical evidence of thrombotic microangiopathy on the background of oliguric AKI, proteinuria and hematuria had prompted the kidney biopsy. Surprisingly no evidence of thrombotic microangiopathy or significant pigmented tubular casts was found. Interestingly, the patient had no evidence of leukocyturia and no significant eosinophilia prior to biopsy, however significant eosinophilia was observed on the day of biopsy (Table 1). The patient had no prior history of medication allergies or skin rashes, but histopathologic findings of skin biopsy were in concordance with an allergic, drug-induced skin reaction (Table 2). Consistent with the possibility of a drug-induced etiology, AKI developed at the same time as the skin rash. The rash had been presumed to be related to Amoxicillin, which had been discontinued. However our patient subsequently received various other beta-lactam antibiotics as illustrated in Fig. 1. Skin rashes are common in patients with COVID-19, with maculo-papular rashes being the most frequent[30]. A drug-induced etiology is often hypothesized as these patients tend to be sicker and thus receive multiple medications compared with patients with other rashes.

The patient described here also developed severe coombs-negative hemolytic anemia with schistocytes on peripheral blood smear. Criteria for thrombocytopenia were not met, but platelet counts did drop by approximately 60%. Work-up excluded thrombotic thrombocytopenic purpura (normal serum-ADAMTS19 activity), paroxysmal nocturnal hematuria or glucose-6-phosphate-deficiency. There was no evidence of hereditary erythrocyte membrane disorder or hemoglobinopathy. An association between hemolytic anemia and interstitial nephritis has been described [31], although in general these cases had a positive Coombs test indicating immune-mediated hemolysis induced by medication. Drug-induced immune-mediated hemolysis with a negative Coombs test, potentially falsely negative due to the severity of the hemolysis and number of transfusions, has however been reported [32]. We were unable to measure specific anti-antibiotic antibodies to test this hypothesis and cannot exclude drug-induced hemolysis. In recent months several cases of auto-immune hemolytic anemias (Coombs positive) in patients with COVID-19 have been described, but most appear to have been associated with underlying diseases and severe AKI was not reported. Of note direct association with COVID itself was postulated in 2 cases [33–35]. Importantly, most of these patients responded favorably to steroid therapy, as did the patient reported here. The presence of schistocytes in the peripheral blood smear in our patient suggests the presence of a microangiopathy, which we were not able to detect in the kidney biopsy. As SARS-CoV-2 infection may be associated with endothelial injury [20, 25], however, the hemolysis may have reflected microvascular injury elsewhere.

GIN is rarely observed in kidney biopsies (< 1% of native kidney biopsies), and the differential diagnosis is broad and challenging [36]. Apart from the usual suspects including medications (especially antibiotics and nonsteroidal anti-inflammatory drugs) and autoimmune disorders (i.e. vasculitis, especially granulomatosis with polyangiitis, sarcoidosis, tubulointerstitial nephritis with uveitis (TINU)-syndrome), microorganisms such as mycobacteria and fungi have been implicated [37]. We could not find evidence of these diseases in the current case. In the case presented here, tuberculosis was excluded with negative cultures and autoimmune disorders were excluded with negative serologies. Sarcoidosis could not be completely ruled out, but given the lack of sharply defined granulomas in the biopsy and in the absence of Schaumann bodies, the histology was most consistent with a drug-induced cause for the GIN. A follow-up serum calcium after hospital discharge, when the patient was no longer on steroids remained within the normal range. Myelin bodies described in the biopsy were sparse, not consistent with a diagnosis of Fabry’s Disease, and were more likely associated with hydroxychloroquine or amiodarone use. Both medications were discontinued. A further differential diagnosis of GIN in our patient included secondary hemophagocytic lymphohistiocytosis (sHLH), which has been associated with COVID-19 [38]. Also known as macrophage activation syndrome, it is a systemic inflammatory syndrome, manifest by a fulminant hypercytokinemia [20, 39, 40]. The clinical picture is broad including fever, hepatosplenomegaly, hepatobiliary dysfunction and pulmonary involvement (including ARDS). Renal injury and cutaneous rash – as present in our patient – may also occur [39]. Laboratory abnormalities include cytopenias, coagulopathy, altered liver function test, hypertriglyceridemia and hyperferritinemia [39]. A bone marrow aspirate was not performed, but given the multiorgan dysfunction and the very high ferritin levels sHLH could not be entirely excluded. Drug reaction with eosinophilia and systemic symptoms (DRESS) syndrome associated with hydroxychloroquine or azithromycin has been reported in a patient with COVID-19 [41]. This patient had mild renal dysfunction and responded to corticosteroid therapy. DRESS syndrome was unlikely in our patient however, given the absence of significant eosinophilia and only transient elevation in liver enzymes.

Taken together, a medication-related etiology of GIN leading to AKI, and possibly to hemolysis and the skin rash, seems most likely here. Whether and how the background inflammatory milieu of COVID-19 might have modulated the disease phenotype or independently contributed to the findings remains unclear. The rapidity of the clinical response in terms of improvement of kidney function and hemolysis suggests a benefit from corticosteroid therapy in this patient. At the time of treatment, corticosteroid therapy was not routinely recommended in COVID-19, and there was even some hesitation about their use. The kidney biopsy findings however prompted in-depth multi-disciplinary discussion and re-review of all the clinical findings and led to a decision to initiate corticosteroid therapy.

Interstitial infiltrates have not commonly been described in the published kidney biopsy series from patients with COVID-19 [8, 10, 13]. As most patients with severe COVID-19 in the ICU likely receive multiple medications known to be associated with interstitial nephritis, this finding may be somewhat surprising. Discussion of the risk of drug reactions in the literature has thus far focused on potential specific therapeutic agents for COVID-19 itself [17, 18], although many other medications are used simultaneously given the severity of illness (Fig. 1). The risk of medication-associated adverse reactions may therefore be more clinically relevant than recognized. Based on the findings in this case, we suggest that this diagnosis should be considered more frequently as a potential indication for a kidney biopsy as there may be important therapeutic consequences.

Given the clinically unexpected finding of GIN in this case and the favorable response to treatment, we suggest that nephrology consultation and kidney biopsy are of value in better understanding the pathophysiology of renal involvement in patients suffering from SARS-CoV2 infection. Even late in the course a kidney biopsy may lead to changes in therapy which can positively impact outcomes.

## Data Availability

Data are displayed in the text, tables and figures. The raw data are available from the corresponding author on reasonable request.

## Abbreviations

COVID-19: Coronavirus disease 19
AKI: Acute kidney injury
SARS-CoV-2: .
ATI: Acute tubular injury
ICU: Intensive care unit
CT: Computed tomography
ARDS: Acute respiratory distress syndrome
G6PD: Glucose-6-phosphate dehydrogenase
CVVHDF: Continuous veno-venous hemodiafiltration
DRESS: Drug reaction with eosinophilia and systemic symptoms
sHLH: Secondary hemophagocytic lymphohistiocytosis
GIN: Granulomatous interstitial nephritis
CRP: C-reactive proteine
PCT: Procalcitonin
IL-6: Interleukin 6
AST: Aspartate amino transferase
ALT: Alanine aminotransferase
LDH: Lactate dehydrogenase
WBC: White blood cells
PCR: Protein/creatinine ratio
ACR: Albumine/creatinine ratio
